# Anti-Restriction Gene Homologs Are Highly Represented in Methicillin-Resistant and Multidrug-Resistant *Staphylococcus aureus* ST239 and ST398: Implications for Resistance Gene Acquisitions

**DOI:** 10.3390/antibiotics11091217

**Published:** 2022-09-08

**Authors:** Deborah Nascimento Santos Silva, Cristiana Ossaille Beltrame, Ana Maria Nunes Botelho, Caroline Lopes Martini, Matheus Assis Côrtes Esteves, Isabella Alvim Guedes, Laurent Emmanuel Dardenne, Agnes Marie Sá Figueiredo

**Affiliations:** 1 Instituto de Microbiologia Paulo de Góes, Centro de Ciências da Saúde, Universidade Federal do Rio de Janeiro, Avenida Carlos Chagas Filho 373, Cidade Universitária, Rio de Janeiro 21941-902, RJ, Brazil; 2 Laboratório Nacional de Computação Científica, Ministério de Ciências, Tecnologia e Inovações, Avenida Getúlio Vargas 333, Quitandinha, Petrópolis 25651-075, RJ, Brazil

**Keywords:** MRSA, gene transfer, antimicrobial resistance, *ardA* homolog, multiresistance

## Abstract

Multidrug resistance is commonly acquired by transferring DNA from one bacterium to another. However, the mechanisms that enhance the acquisitions of foreign genes are poorly understood, as well as the dynamics of their transmission between hosts in different environments. Here, genomic approaches were applied to evaluate the enrichment of the *S. aureus* chromosome with resistance traits in groups of genomes with or without anti-restriction genes and to analyze some evolutionary aspects of these acquisitions. Furthermore, the role played by an anti-restriction gene in improving multiresistance in MRSA was investigated by molecular cloning. A strong association was observed between the presence of anti-restriction gene homologs and patterns of multidrug resistance. Human isolates, mainly ST239-SCC*mec*III, carry *ardA*-H1, and from animal sources, mainly CC398, carry *ardA*-H2. Increased DNA transfer was observed for clones that express the *ardA*-H1 allele, corroborating its role in promoting gene transfer. In addition, *ardA*-H1 was expressed in the dsDNA format in the BMB9393 strain. The evolution of successful multidrug-resistant MRSA lineages of the ST239 and ST398 was initiated not only by the entry of the *mec* cassette but also by the acquisition of anti-restriction gene homologs. Understanding the mechanisms that affect DNA transfer may provide new tools to control the spread of drug resistance.

## 1. Introduction

Antimicrobial resistance (AMR) is a global threat to human health. Methicillin-resistant *Staphylococcus aureus* (MRSA) plays an important role in both hospital-associated (HA-MRSA) and community-acquired (CA-MRSA) infections worldwide [1]. In 2017, the World Health Organization (WHO) categorized MRSA as a high priority microorganism for the development of new antimicrobial drugs [2]. MRSA strains can also infect animals, and the zoonotic transmission of livestock-associated MRSA (LA-MRSA) of the ST398(CC398)-SCC*mec*V lineage is of concern [3]. In humans, LA-MRSA can be associated with severe diseases, including bacteremia and necrotizing infections [3]. It is alarming that the vast majority of ST398-SCC*mec*V (>80%) are resistant to multiple types of antibiotics [4]. Strains from the ST239(CC8)-SCC*mec*III lineage are other important high-level multidrug-resistant MRSA; however, unlike the LA-MRSA, ST239-SCC*mec*III is a typical globally spread HA-MRSA [1].

Despite the importance of AMR, the transmission dynamics of resistant traits in different hosts and environments has not been fully described [5]. An understanding of the mechanisms involved in the acquisition of resistance genes would provide new insights into how multidrug resistance emerges, evolves, and is transmitted. For some bacterial species, including *S. aureus,* horizontal gene transfer (HGT) is a key mechanism leading to bacterial diversification and evolution. HGT is generally mediated by mobile genetic elements (MGEs), including plasmids, transposons, and genomic islands, among others. The transfer of these elements leads to the spread of genes, including antimicrobial resistance genes [1,5]. However, bacteria have mechanisms to prevent exogenous DNA from being incorporated into their genomes.

The restriction-modification systems have two main components, a restriction enzyme that fragments unmethylated DNA and a methylase, which methylates the DNA of the bacterial cell that has the system. Consequently, the restriction enzyme cannot cut the methylated DNA of the bacterial cell. Experimentally, it has been shown that the role of RM systems is to protect the bacterial cell against invasion of foreign DNA. On the other hand, foreign DNA can also carry mechanisms that oppose the bacterial defenses. Example of these mechanisms are the presence of *ardA* genes (alleviation of restriction of DNA) in MGEs, absence of specific sites for the restriction enzymes in the exogeneous DNA, among others [6,7].

Studies on *E. coli* have shown that the ArdA protein and its homologues mimic double-stranded DNA (dsDNA) by imitating the B structure of the dsDNA. Due to this spatial structure, anti-restriction proteins compete with the DNA binding site of the restriction modification type I system (RM I system), impairing DNA degradation and methylation, and thus facilitating HGT [6,7]. The *ardA* homologs are not only distributed within species but can also be transferred between species that have functional RM systems [6]. As HGT is an important gene acquisition mechanism, anti-restriction mechanisms may be also involved in the spread of antimicrobial resistance. In this work, we searched for, localized, and characterized *ardA* homologs in *S*. *aureus* and other Gram-positive bacteria. Our data show that this gene is a biomarker of multidrug resistance for *S. aureus* and suggest that *ardA* homologs may possibly play a role in the acquisition of high-level multidrug resistance by the MRSA strains from ST239 and ST398 lineages.

## 2. Results and Discussion

**Figure 1:**
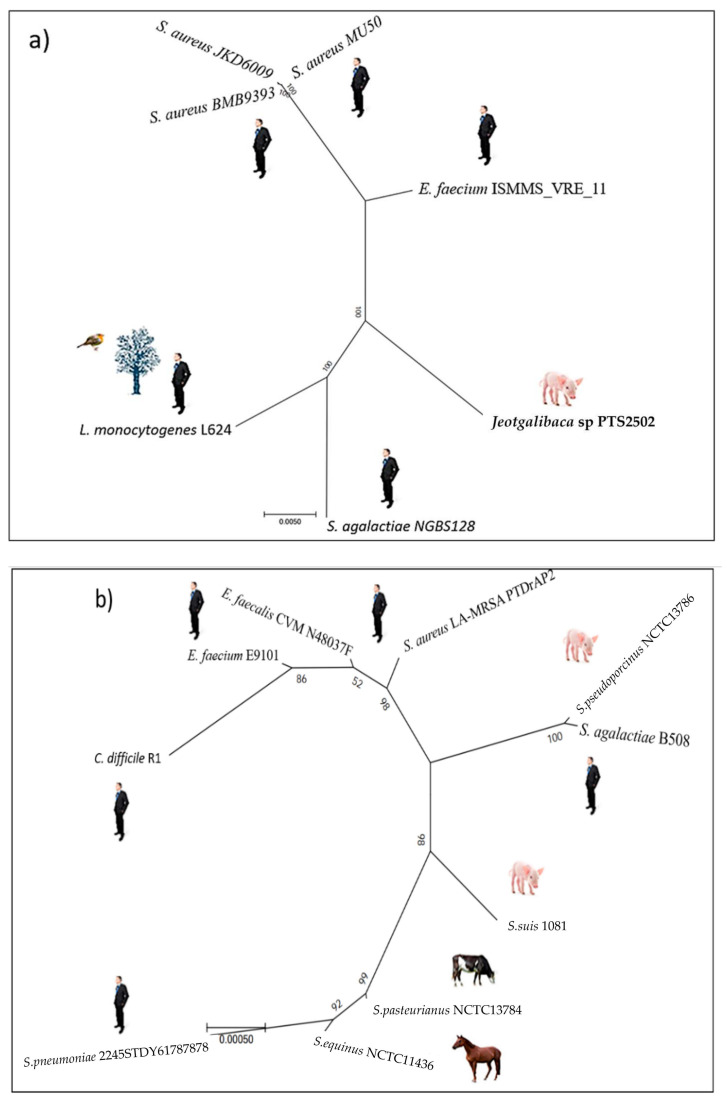
Maximum likelihood tree for *ardA* homologs of selected Gram-positive species illustrating the distribution of *ardA* alleles amongst Gram-positive bacteria. (**a**) *ardA*-H1 homologs. (**b**) *ardA*-H2 homologs.

**Distribution of the *ardA-*H1 allele.***ardA*-H1 is carried by a copy of the Tn*5801* inserted into the bacterial chromosome in all the strains examined. This allele is conserved in different *S. aureus* lineages found in nosocomial infections, as well as in other Gram-positive species, represented mainly by *E. faecium*. For example, the first 250 genomes listed in the “bacteria” database included the HA-MRSA Mu50 (the archetype of the USA100 clone (ST5(CC5)-SCC*mec*II), Acc: BA000017, 100% coverage, 100% identity); HA-MRSA JKD6009 (closely related to the BMB9393 strain (ST239(CC8)-SCC*mec*III), Acc: LR027876, 100% coverage, 100% identity); *E. faecium* ISMMS_VRE_11 (Acc: CP016163, 100% coverage, 100% identity); and *Streptococcus agalactiae* NGBS128 (Acc: CP012480, 98.00% coverage, 94.93% identity); in addition to other species of Gram-positive bacteria commonly found in human infections. Importantly, anti-restriction genes with a high nucleotide identity to *ardA*-H1 were also found in Gram-positive cocci from animals, such as *Jeotgalibaca* sp PTS2502 (Acc: CP019433, 100% coverage, 95.21% identity) isolated from pigs [8,9], as well as *Listeria monocytogenes* L2624 (Acc: CP007686.1, 98.00% coverage, 94.93% identity), a human pathogen that can be isolated from various environmental sources [10] (Figure 1a, Supplementary File S1). As most of the MRSA analyzed are often collected from humans, and the *ardA*-H1 allele is the main *ardA* homolog among human isolates, this allele was first recognized among *S. aureus*. However, toward the end of this study, a second *ardA*-H allele was detected when MRSA isolated mainly from animals was included in this study. This new allele was called *ardA*-H2.

***ardA-*H2 is widely spread in livestock MRSA (LA-MRSA).** The use of Tn*916* from *E. faecalis* DS16 as a reference led to the discovery of the allele *ardA-*H2, which was primarily found among LA-MRSA. Therefore, the reference sequence chosen to represent ArdA-H2 was the amino acid sequence (locus_tag: DD562_05595) carried by a Tn*916* copy inserted into the chromosome sequence of the LA-MRSA strain PTDrAP2 (Acc: CP029172), which belongs to the ST398(CC398)-SCC*mec*V lineage.

**Figure 2:**
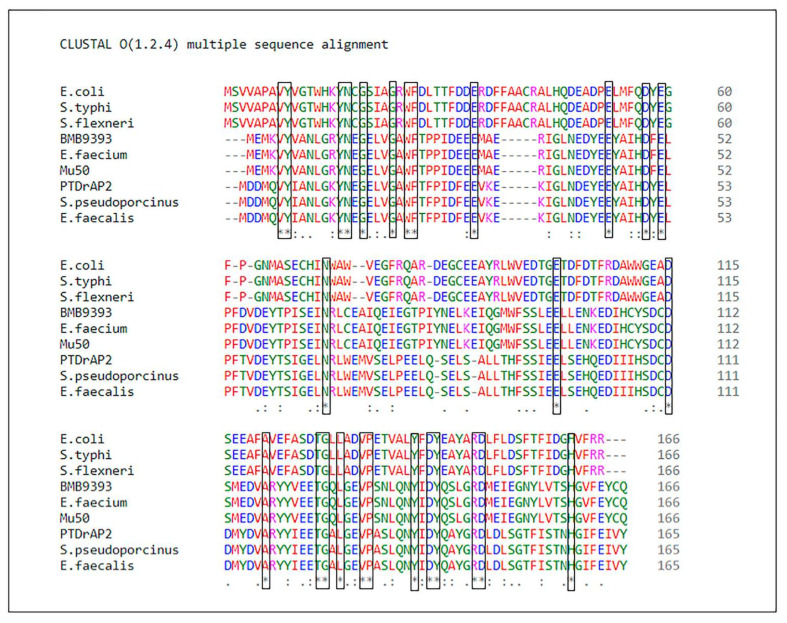
Alignment of ArdA-H amino acid sequences of different Gram-positive and Gram-negative bacteria. E. coli: *E. coli* strain SE11 (Acc: B6IC30); S. typhi: *Salmonella typhimurium* strain 4/74 (Acc: E8XLS4); S. flexneri: *Shigella flexneri* (Acc: A0A2A2XIH3); BMB9393: MRSA strain BMB9393 (Acc: WP_000425404); E. faecium: *E. faecium* ISMMS_VRE_11 (Acc: AON59648.1); Mu50: MRSA strain Mu50 ATCC 700699 (Acc: A0A0H3JPP7); PTDrAP2: LA-MRSA strain PTDrAP2 (Acc: AWI95998.1); S. pseudoporcinus: *Streptococcus* *pseudoporcinus* (Acc: VEF93293.1); E. faecalis: *E. faecalis* DS16 (Acc: U09422.1). Red: Small [small + hydrophobic (incl. aromatic-Y)]; blue: acid; green: hydroxyl + amino + basic − Q; pink: basic; (* and **): unambiguously aligned amino acids; (-): gap; (.): semi-conservative substitution; (:): conservative substitution (): non-conservative substitution.

In addition to its presence in LA-MRSA ST398-SCC*mec*V, *ardA-*H2 was conserved in a variety of Gram-positive bacteria (100% coverage, 100% nucleotide identity), including bacteria of clinical importance to humans, such as *Streptococcus pneumoniae* 2245STDY6178787 (Acc: LR216060) and *S. agalactiae* B508 (Acc: CP021770). Furthermore, Gram-positive species found in livestock animals besides LA-MRSA can carry an identical *ardA-*H2, for example, *Streptococcus pseudoporcinus* strain NCTC13786 (Acc: LR134341), *Streptococcus equinus* strain CNU G6 (Acc: CP046629) and *Streptococcus suis* strain 1081 (Acc: CP017667) (Figure 1b, Supplementary File S1). ArdA-H2 of the PTDrAP2 strain exhibited 63.35% amino acid identity (98% coverage, e-value: 3e-74) with ArdA-H1 of the HA-MRSA strain BMB9393. The alignment of the amino acid sequences of these two alleles in different Gram-positive bacteria, and also of *E. coli* ArdA and its homologs in other Gram-negative species, showed conserved residues that were aligned unambiguously, as expected in homologous alignments (Figure 2).

**Figure 3:**
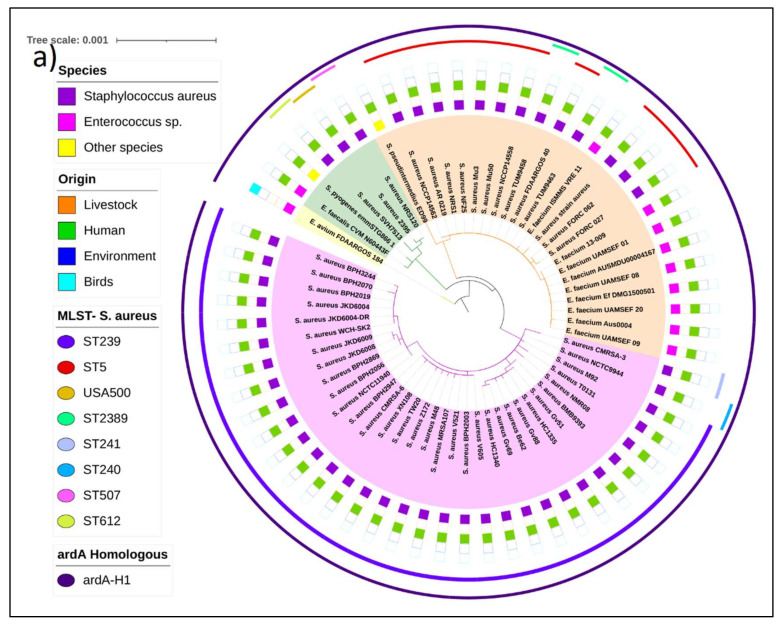

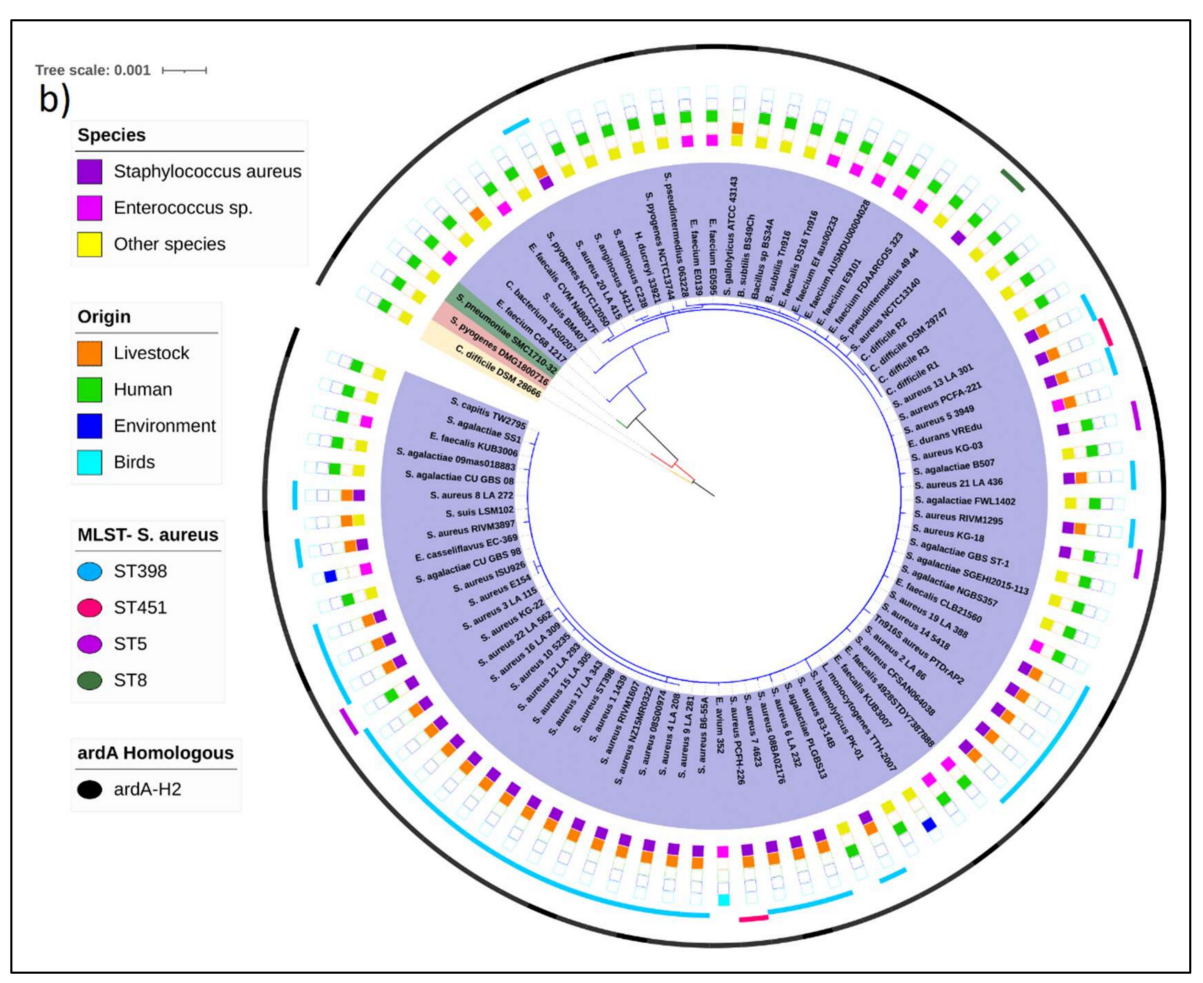
Maximum likelihood phylogenetic tree constructed based on SNP calling, using the nucleotide sequence of Tn*5801* (**a**) and Tn*916* (**b**) from Gram-positive bacteria. The innermost rectangles represent bacterial species, and the outermost represent the origin. The innermost lines represent the sequence type of the multilocus sequence typing, and the outermost lines represent the presence of *ardA*-H1 and ardA-H2.

**Phylogenetic trees of the Tn*916* family of transposons carrying the *ardA*-H1 and *ardA*-H2 alleles.** The estimated maximum likelihood (ML) tree topology for Tn*5801* transposon carrying *ardA*-H1 revealed three main independent clades. The more basal genomes were from *Enterococcus avium*, *E. faecalis*, and *Streptococcus pyogenes,* followed by three genomes of *S. aureus*. It is possible that the origin of Tn*5801* from *S. aureus* is in the group of catalase-negative, Gram-positive cocci. The Tn*5801* sequence of the HA-MRSA strain Mu50 and related genomes clustered in the orange clade (Figure 3a). Intra-genus horizontal transfer of Tn*5801* from MU50-related genomes to some strains of *E. faecium* is suggested by the topology of this tree (Figure 3a, orange clade).

Another clade (pink) grouped the Tn*5801* sequence of the strain BMB9393 and other ST239 genomes from different countries, supporting the hypothesis that the acquisition of *ardA*-H1 (carried by this transposon) by ST239 strains occurred before the adaptive radiation and spread of this lineage to different continents [1].

An ML tree was also constructed for the Tn*916*, which carries the *ardA*-H2 allele. The most basal sequences in this tree are from *Clostridioides difficile*, *S. pyogenes,* and *S. pneumoniae*. It is notable that the Tn*916* from PTDrAP2 clustered with the transposon sequences carried by other LA-MRSA (ST398) and also by *S. agalactiae*, *E. faecalis*, *Staphylococcus pseudintermedius*, *C. difficile*, and *Bacillus subtillis*, in addition to other Gram-positive species. León-Sampedro and colleagues [11] also detected highly conserved Tn*916* elements integrated into the genomes of different Gram-positive bacteria. Taken together, these data suggest recent transmissions of *ardA*-H2 between animal and human strains of a range of Gram-positive bacterial species, which is consistent with the unconstrained bacterial promiscuity promoted by *Tn916* [11]. In contrast to Tn*5801,* whose transfer appears to be more restrictive, the tree topology for Tn*916* suggests a more promiscuous transfer for the latter transposon (Figure 3b). Despite this, our analysis suggested the occurrence of intergenus transfer of Tn*5801* from *S. aureus* to *E. faecium* (Figure 3a). In fact, vancomycin resistance arose in a MU50-related strain of MRSA [12], which was also found here as *ardA*-H1 (+). In some reported cases, the vancomycin-resistant *S. aureus* (VRSA) emerged during co-infection with vancomycin-resistant *Enterococcus* (VRE), suggesting that gene transfers from *Enterococcus* sp to *S. aureus* can also occur in vivo during natural infections [13,14]. Thus, all these data suggest that the exchange of genetic material between *S. aureus* and *Enterococcus* sp can occur in both directions.

**Figure 4:**
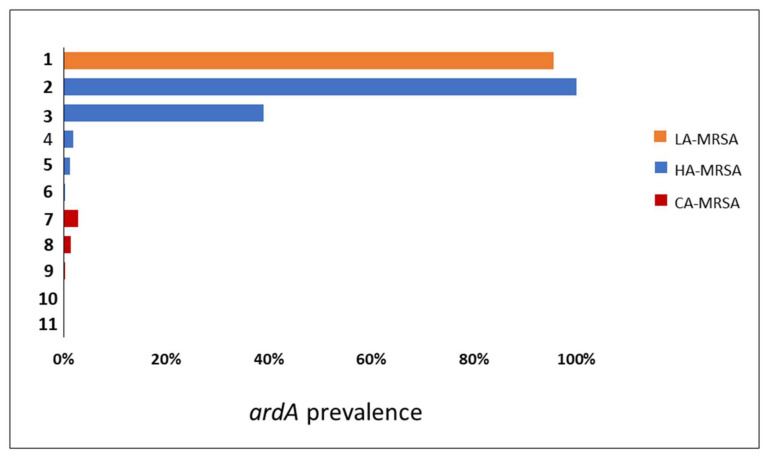
Assessment of *ardA*-H1 and *ardA*-H2 in the *S. aureus* local database composed of genomes that display MLST clonal complexes (CCs) commonly found among hospital-associated MRSA (*n* = 3052); genomes from CCs typically found in CA-MRSA (*n* = 2384) and genomes from CCs typically found in LA-MRSA (*n* = 780). These genomes were related to the following *S. aureus* lineages: HA-MRSA; (**1**) ST398-SCC*mec*V (LA-MRSA clone); (**2**) ST239-SCC*mec*III (Brazilian clone; HA-MRSA); (**3**) ST8-SCC*mec*IV (USA500 clone, HA-MRSA); (**4**) ST5-SCC*mec*II (USA100 clone, HA-MRSA); (**5**) ST45-SCC*mec*II (USA600 clone, HA-MRSA); (**6**) ST22-SCC*mec*IV (MRSA-15 clone, HA-MRSA); (**7**) ST30-SCC*mec*IV (USA1100 clone, HA-MRSA); (**8**) ST1-SCC*mec*IV (USA400 clone, HA-MRSA); (**9**) ST8-SCC*mec*IV (USA300 clone, HA-MRSA); (**10**) ST80-SCC*mec*IV (European clone, HA-MRSA); and (**11**) ST59-SCC*mec*V (Taiwan clone, HA-MRSA).

**Figure 5:**
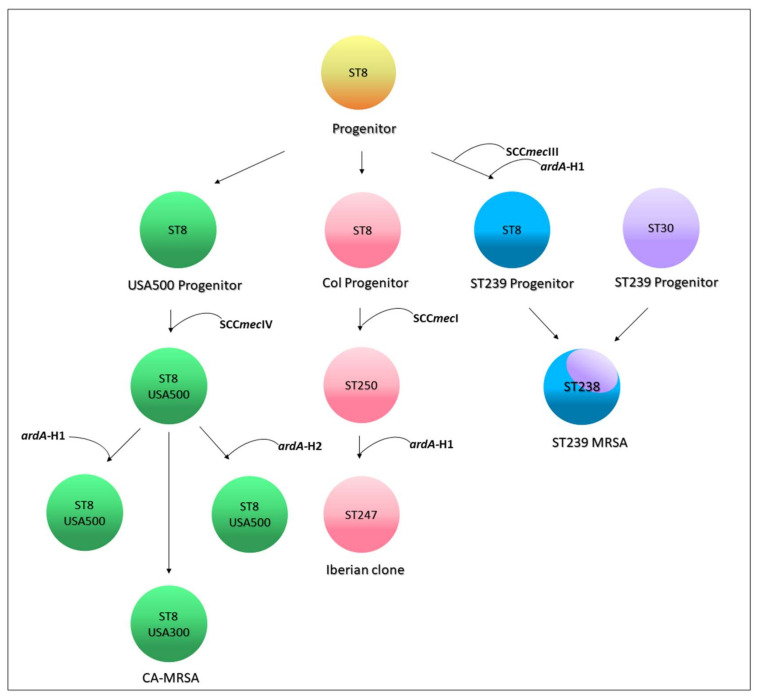
Genetic evolution of *ardA-*H1 gene in the complex clonal 8 (CC8). Note: unlike USA500 MRSA, the entry of *ardA*-H1 into the ST239 genome may have occurred prior to ST239 differentiation. ST239 MRSA is a hybrid of the ancestors ST8 and ST30.

**Searching for *ardA-*H in contemporary MRSA lineages.** The search for *ardA-*H1 in 6215 *S. aureus* genomes showed that this allele was most associated with CC8 genomes of HA-MRSA, including those related to multidrug-resistant strains of the ST239(CC8)-SCC*mec*III lineage (*n* = 224/224; 100%) and to HA-MRSA strains named USA500 (*n* = 132/339; 39%) (Figure 4). Based on the evolution of ST8 [1,15], and the analyses performed here, we proposed that the *ardA-*H1 was introduced into a CC8 genome ST239 progenitor probably before the ST239 diversification and worldwide spread of this HA-MRSA lineage [1]. This model is also supported by the fact that all the ST239 genomes deposited in the GenBank carry *ardA*-H1. However, in USA500(CC8)-related genomes, *ardA* appears to have entered after USA500 diversification, because genomes that lack *ardA*-H, contain *ardA*-H1, and a few with *ardA*-H2 were found in this study (Figure 5). In addition to CC8, *ardA-*H1 was detected at a lower frequency among CC5, CC22, and CC45 genomes. The *ardA-*H2 allele, however, was almost universally found in strains related to LA-MRSA of the ST398 lineage (*n* = 746/780; 95.64%) (Figure 4). Likewise, this gene was present in the genomes of other ST analyzed related to ST398, including ST3783, ST3226, ST3709, ST752, ST3706 and ST541.

Notably, *ardA*-H (mainly *ardA*-H1) was found at a very low frequency in the 2384 genomes that belong to CCs commonly associated with CA-MRSA (*n* = 16/2384; 0.34%), including those related to the clones USA300 (CC8; *n* = 2/1447; 0.14%), USA400 (CC1; *n* = 2/157; 1.27%), and USA1100 (CC30; *n* = 12/438; 2.74%) (Figure 4; *p* < 0.0001). Among the genomes related to HA-MRSA, the prevalence of *ardA*-H was 12.4% (*n* = 379/3052). The presence of *ardA*-H1 was particularly detected among CC8 MRSA-related genomes (ST239-SCC*mec*III and USA500), strains classically involved in nosocomial infections and known to present high-level multiresistance (356/563; 63.2%) (Figure 4). It can be argued that the high number of resistance traits found in these hospital-associated CC8 genomes (ST239-SCC*mec*III and USA500) could be due to the intrinsic characteristics of the CC8 background and not to the presence of *ardA*-H1 in the bacterial chromosome. However, contrary this assumption is the fact that among the genomes (*n* = 1447) of the ST8 (CC8) strains related to the highly susceptible CA-MRSA, USA300 [16], only two genomes carry *ardA*-H2.

**Figure 6:**
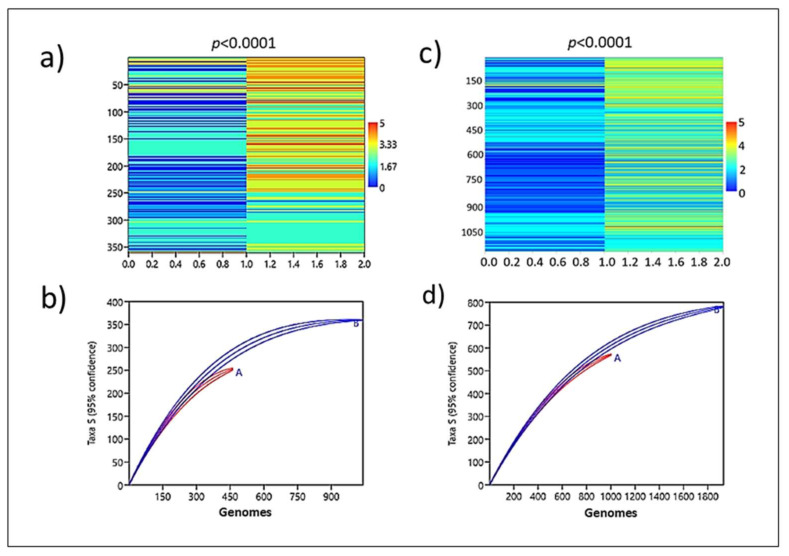
Interpolated plot from the transition matrix, showing the enrichment of antimicrobial resistance genes among *ardA*-H positive (−) in comparison with *ardA*-H negative (−) genomes. (**a**) A total of 720 genomes were analyzed with the following distribution: *ardA*-H (−) (*n* = 360) and *ardA*-H (+) (allele-1 (*n* = 360)). (**b**) A total of 1564 genomes were analyzed with the following distribution: *ardA*-H (−) (*n* = 782) and *ardA*-H2 (+) (*n* = 782). Color scale: 0 = susceptibility to all antimicrobial drugs, except β-lactams; 1 = resistance to one; 2 = two; 4 = four; and 5 = five or more antimicrobial classes. (**c**,**d**): Individual rarefaction, showing the distribution of resistance traits in *S. aureus* genomes with 95% confidence intervals. (**c**) Population A (red plot) is formed by genomes lacking *ardA*-H (*n* = 360) and population B (blue plot) is formed by genomes carrying *ardA*-H (+) (allele-1(*n* = 360)). (**d**) Population A (red plot) is formed by genomes lacking *ardA*-H (*n* = 782), and population B (blue plot) is formed by genomes carrying *ardA*-H2 (*n* = 782).

**Analysis of the enrichment of the resistance gene.** Corroborating a role played by *ardA*-H in the acquisition of resistance genes, the genomic group formed by *ardA-*H1 (+) accumulated significantly more resistance traits (*p* < 0.0001) compared to that formed by *ardA-*H (−) (Figure 6a,b; Supplementary File S2). Again, supporting these data, a strong association between multidrug resistance and the presence of *ardA-*H2 in these genomes was also detected. Likewise, the enrichment analysis shows that resistant genes are clearly over-represented in *ardA-*H2 (+) compared to *ardA*-H (−) genomes (Figure 6c,d). Thus, these data indicate an increased acquisition of antimicrobial-resistant traits in *ardA*-H1 and *ardA*-H2 populations of *S. aureus*.

**Figure 7:**
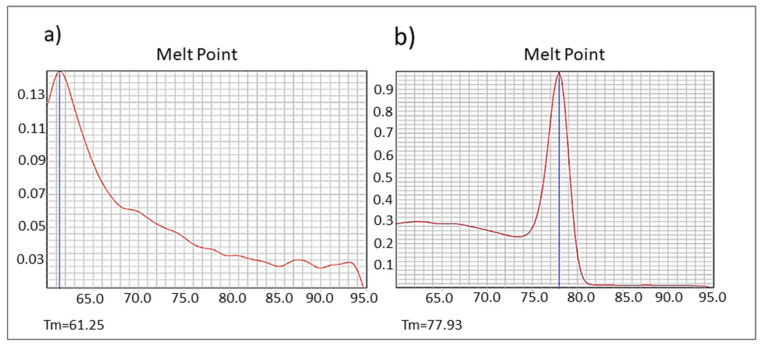
Expression of *ard*A-H1 in the strain BMB9393 in dsDNA format. (**a**) Melting plot for strain CR15-051 that does not carry *ard*A-H alleles, showing the expected melting curve for *ardA*-H1 primers (61–62 °C). (**b**) Melting plot for strain BMB9393 carrying *ard*A-H1 in the ICE Tn*5801,* showing the expected melting curve for *ard*A-H1 cDNA (77–78 °C) using specific *ard*A-H1 primers.

***ardA*-H1 expression as dsDNA.** Some anti-restriction genes, such as the *E. coli ardC* gene, are expressed in the recipient strain as dsDNA; also, the protein is not exported from the donor to the recipient cells, as demonstrated by González-Montes et al. 2020 [17]. Therefore, this mechanism may increase gene acquisition in the bacterial hosts. Those authors also suggested that inhibition of ArdC activity could provide a new tool to hinder the transmission of multidrug resistance [17]. However, other authors have hypothesized that *ardA* could be transcribed from single-stranded (conjugative) DNA (ssDNA), prior to the synthesis of complementary DNA in the recipient strain, possibly due to the presence of specific secondary structures formed by ssDNA [18]. As also demonstrated for *ardC* [17], here, we show that the *ardA*-H1 naturally present in the genome of the BMB9393 was expressed in the form of dsDNA (Figure 7a,b).

**Figure 8:**
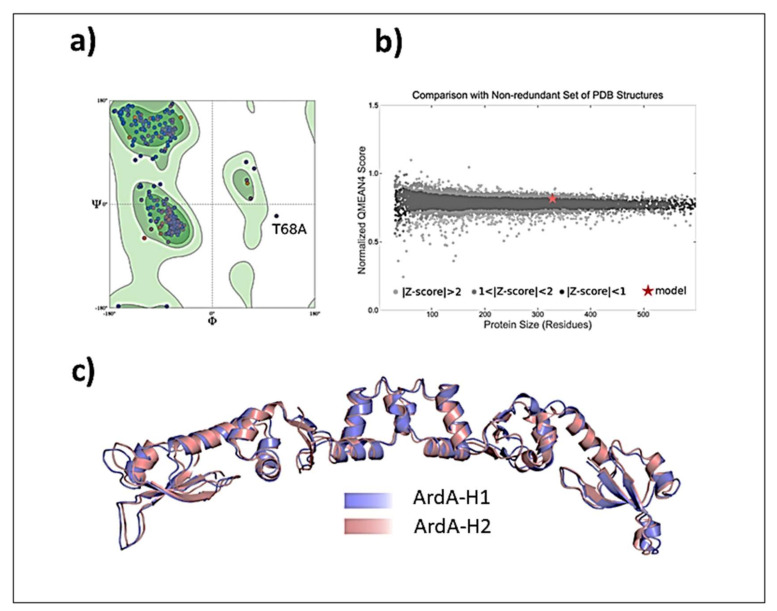
(**a**) MolProbity Ramachandran plot with the outlier residue Thr78 from chain A located outside the allowed regions. (**b**) QMEANDisCo plot. (**c**) ArdA-H1 (purple) superposed with the ArdA-H2 structure used as a template (PDB code 2W82, colored salmon).

**Figure 9:**
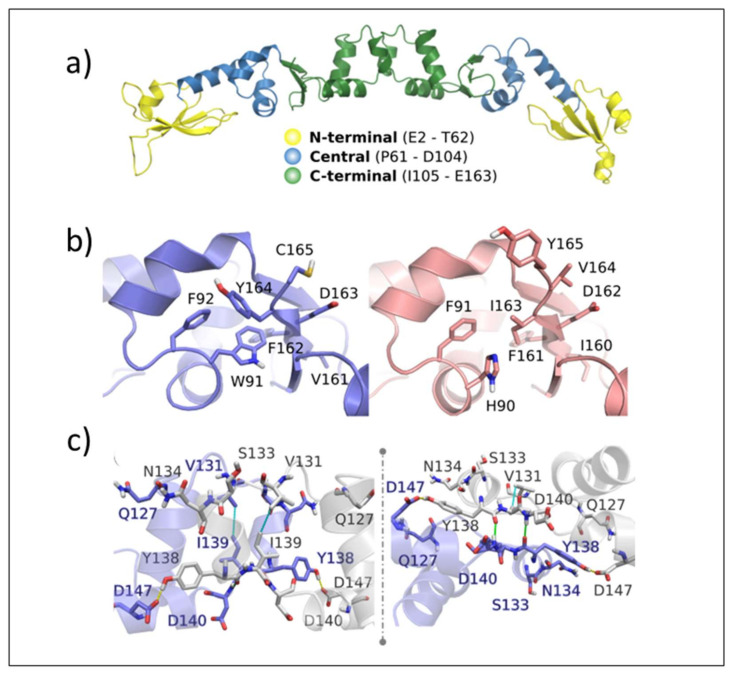
(**a**) ArdA-H1 domains: N-terminal (yellow), central (blue), and C-terminal (green). (**b**) Essential residues and the VF-motif (V161 and F162) of ArdA-H1 (left, purple) and ArdA-H2 (right, salmon). (**c**) The ArdA-H1 anti-restriction domain with the main amino acids represented as sticks and rotated 45° (right). Chain A (purple) and chain B (grey). Hydrogen bonds between side chains and main chains are highlighted in yellow dashed and green solid lines, respectively. Nonpolar interactions are highlighted in cyan dashed lines.

**Validation of ArdA-H1 model.** The analyses performed with MolProbity indicated that the model constructed for ArdA-H1 presented 96.91% of all residues in favored regions and only one outlier residue was located in the central domain (Thr78 from chain A) (Figure 8a). The QMEANDisCo result shows good energetic and geometric quality, with an overall score of 0.79 ± 0.05 (Figure 8b). Due to the high similarity between the protein sequences, ArdA-H1 shares the same folding of ArdA-H2 with preserved secondary structures (Figure 8c). ArdA-H1 shares the same 3D arrangement of ArdA-H2 with each monomer of the dimeric structure containing the following three domains: (i) N-terminal (E2-T62), (ii) central (P61-D104), and (iii) C-terminal (I105-E165) (Figure 8a). The most important amino acid residues for ArdA-H2 are listed in Supplementary Table S1. These analyses focused on the central and C-terminal domains because previous work revealed that the N-terminal domain is not important for the anti-restriction activity, as it can be deleted without affecting the binding against the MTase core [19]. The residues previously described as essential for the structural integrity of the ArdA-H2 protein and the anti-restriction activity [6,19] are highly conserved between the ArdA-H1 and ArdA-H2 structures (Figure 9a,b). In the anti-restriction motif (Figure 9c), ArdA-H1 contains residues Gln127, Ser133, and Asn134 instead of Ala126, Ala131, and Ser133, respectively, in ArdA-H2 (not shown). These residues are exposed to solvents in both structures, and their presence in ArdA-H1 might provide additional polar (e.g., hydrogen bonds) interactions during the molecular recognition process. Despite these differences, the high sequence and structural similarities of those proteins suggest that these alleles are likely to be active in vivo.

**Figure 10:**
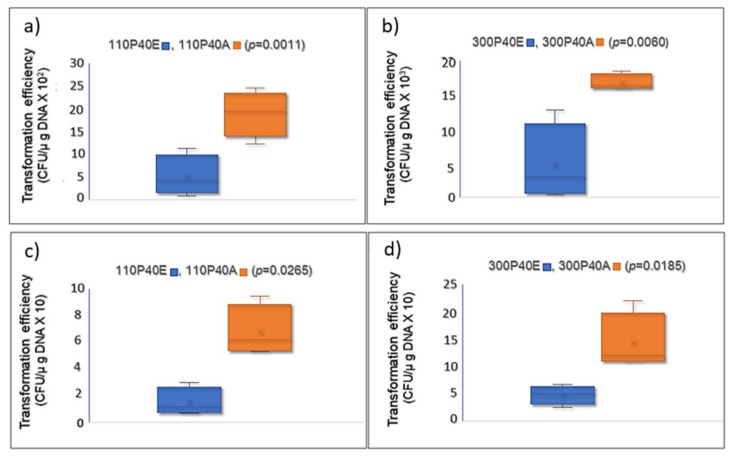
Transfer of exogenous DNA to *S. aureus* clones 110P40A and 300P40A (*ardA*-H1 (+)), and the isogenic strains 110P40E and 300P40E (*ardA*-H1 (−)). (**a**,**b**) pBMB9393, a natural plasmid obtained from *S. aureus* and (**c**,**d**) pLI50, plasmid vector obtained from *E. coli*.

**ardA-H1 effectively increases exogenous DNA incorporation into *S. aureus* cells.** The amino acid sequence of the *E. faecalis ardA* homolog (Tn*916*, ORF 18) [6] is identical to that of the ArdA-H2 allele found in LA-MRSA (Figure 2). In fact, this homolog was effective in blocking restriction activities in vivo when ORF18 was cloned in *E. coli* that expressed different type I RM families [6]. Furthermore, ArdA-H2 has the same 3D arrangement and overlaps with the experimentally defined structure of ArdA ORF18. Thus, we chose to clone the *ardA*-H1 gene from BMB9393, a hospital strain of the ST239(CC8)-SCC*mec*III lineage, one of most important MRSA in human infections, to test the effect of *ardA*-H1 on gene transfer using two clinical CA-MRSA strains of different genetic backgrounds. Our data clearly show increased acquisition of DNA for the clones that expressed *ardA-*H1. The rates of *cat* gene acquisition (carried by pBMB9393) by strains 110P40A and 300P40A, both expressing ArdA*-*H1, were increased 3.67- and 3.53-fold, respectively, compared with 110P40E and 300P40E, the corresponding isogenic *ardA-*H1 (−) strains (Figure 10a,b). A similar increase (3.00–3.53 folds) in DNA transfer was observed when heterologous *E. coli* DNA was transformed into isogenic strains (Figure 10c,d).

The rates of *cat* gene acquisition (carried by pBMB9393) by strains 110P40A and 300P40A, both expressing ArdA*-*H1, were increased 3.67- and 3.53-fold, respectively, compared with 110P40E and 300P40E, the corresponding isogenic *ardA-*H1 (−) strains (Figure 10a,b). A similar increase (3.00–3.53 folds) in DNA transfer was observed when heterologous *E. coli* DNA was transformed into isogenic strains (Figure 10c,d). Consistent with these findings, McMahon and colleagues [6] demonstrated the in vitro heterologous activity of ArdA homologs from different bacterial species against type I RM systems, including MRSA Mu50 (allele H1).

However, the *ardA*-H1 gene from MU50 cloned in *E. coli* failed to induce high levels of protein expression [6], and those authors did not demonstrate its role in DNA transfer in *S. aureus*.

Taken together, our study shows that the *ardA*-H1 of the BMB9393 strain is transcribed in dsDNA format and indicates that the ArdA-H1 protein is expressed and active, as demonstrated by the increased transfer of exogenous DNA to pCN40::*ardA-*H1 clones in comparison with isogenic strains *(ardA*-negative), as shown previously for *ardA*-H2 [6,7]. These data are further validated by the spatial sequence similarity between ArdA-H1 and ArdA-H2 proteins, as demonstrated here. Furthermore, we revealed that the genomes of strains ST239 (CC8; HA-MRSA) and ST398 (CC398; LA-MRSA) that carry *ardA*-H alleles (*ardA*-H1 and *ardA*-H2; respectively) are enriched with antimicrobial resistance genes, while these genes were poorly detected in the genomes of *S. aureus* lineages associated with the very susceptible CA-MRSA strains. Therefore, our data indicate that *ardA*-H1 plays a role in promoting gene acquisition in MRSA and, consequently, might influence the dissemination of resistance genes in different environments.

## 3. Material and Methods

**Searching for *ardA* gene in a local *S. aureus* database.** A database, consisting of 6215 genomic sequences, was created in this study to encompass all genomic sequences available in the GeneBank of the most important MLST clonal complexes (CCs) of MRSA circulating in humans and animals (Supplementary File S3, http://data.mendeley.com/datasets/mkwvsp8rhg (published on 19 April 2022)). The two *ardA* homolog alleles detected during this investigation in *S. aureus* were named *ardA*-H1 (501 bp) and *ardA*-H2 (498 pb) and correspond to the nucleotide sequences found in strains BMB9393 (ST239 (CC8)) (Acc: CP005288; locus_tag: SABB_RSO2195) and PTDrAP2 (ST398(CC398)) (Acc: CP029172; locus_tag: DD562_05595), respectively. These sequences were used as references to search for *ardA* homologs in the local *S. aureus* database using basic local alignment search tool (BLAST) command line applications (https://www.ncbi.nlm.nih.gov/books/NBK279690/ (accessed on 3 May 2019)). As we found *ardA*-H1 located in the Tn*5801* transposon, which belongs to the Tn*916* family, the complete nucleotide sequence of Tn*916* of the *Enterococcus faecalis* DS16 (Acc: U09422.1) was also used as a reference sequence for the search for *ardA* homologs in the local database. In addition, BLASTn (https://blast.ncbi.nlm.nih.gov/Blast.cgi (accessed on 3 May 2019)) was run using default parameters in the NCBI databases “bacteria (taxid:2)” and “*Enterococcus* (taxid: 1350)”. The UniProtKB BLAST tool (http://www.uniprot.org/blast/ (accessed on 20 May 2019)) with default parameters was also run to look for BMB9393 anti-restriction protein homologs (Uniprot accession number (UAcc): A0A2 × 2LRV4) in *S. aureus* and other bacterial species. This same application was performed using the *ardA* amino acid sequence of the *E. coli* strain SE11 (UAcc: B6IC30). Subsequently, representative sequences of *ardA* homologs from different Gram-positive and Gram-negative species were selected based on sequence differences. These sequences were aligned using Clustal Omega (1.2.4- https://www.ebi.ac.uk/Tools/msa/clustalo (accessed on 20 May 2019)). The location of the *ardA* homologs in *S. aureus* genomes was examined using IslandViewer 4 with integrated prediction methods [20] and the MicroScope genomic platform [21].

**Phylogenetic trees of transposable elements carrying homologs of *ardA* (*ardA*-H).** To build a phylogenetic tree for Tn*5801*, the sequences were selected by defining a cut-off value of 99.90% nucleotide identity and 91.00% coverage (Supplementary File S4), using as a reference the Tn*5801* sequence of the strain ST239 (CC8) named BMB9393 (Acc: CP005288; coordinates 451.953 to 480.229). These sequences were aligned using REALPHY. The maximum likelihood tree was obtained by applying Mega7 algorithms with default parameters using the Tamura-Nei model with 1000 bootstrap replicates [22]. The Tn*5801* sequence of the strain *E. avium* FDAARGOS 184 (Acc: NZ_CP024590) was used to root the tree. For the phylogenetic tree of Tn*916*, the reference was the nucleotide sequence of Tn*916* of the strain ST398 (CC398) named PTDrAP2 (Acc: CP029172; coordinates: 1,064,554 to 1,082,585), and the same cut-off value was applied for the selection of 41 Tn*916* sequences from *S. aureus* and 48 from other Gram-positive species (Supplementary File S4). The Tn*916* phylogenetic tree was constructed as described. The Tn*916* sequence of the strain *C. difficile* DSM 28666 (ACC: NZ_CP012321) was used to root the tree. The Interactive Tree Of Life (iTOL) v.4 tool was used to visualize the trees [23].

**Gene set enrichment analysis for the resistance genes**. Whole genomes carrying *ardA*-H and an equal number of sequences that lacked these genes were randomly selected from the *S. aureus* local database (Supplementary File S2), using the Kutools for Excel 24.0 (Informer Technologies, Inc). The selected genomes were used to test for a statistical association between the absence/presence of *ardA-*H and the acquisition of multidrug resistance in *S. aureus*. Data on resistant genes were collected using the ResFinder 3.1 database composed only of genes acquired horizontally [24], using threshold values of 90% and a coverage of 90%. This analysis was performed for all 360 *ardA*-H1 and 782 *ardA*-H2 genomes available on GenBank, balanced by the same number of genomes that lacked these genes (*ardA*-H (−) genomes) (Supplementary File S2). The Markov chain transition matrix was used to analyze the process of gene acquisitions in the state transition of *ardA*-H (−) and *ardA*-H (+) genomes. The χ^2^ test was used to calculate the probability that data would be taken from a system with random proportions of transitions (i.e., no preferred state). This analysis was performed using Past software 4.03 [25]. Multidrug resistance was defined as resistance to four antimicrobial drugs, including beta-lactams. High-level multidrug resistance was defined as resistance to more than four antimicrobials, including beta-lactams.

**Three-dimensional (3D) structures of ArdA-H allele-1 and allele-2.** Currently, there is a high-resolution 3D structure of ArdA-H2 in the Protein Data Bank (PDB), which has been solved experimentally (PDB code 2W82, *E. faecalis*) by means of X-ray diffraction with a resolution of 2.8 Å. As no experimental structure of ArdA-H1 is available at PDB, we built the 3D homodimeric structure by comparative modelling with the SWISS-MODEL web server [26,27], available at https://swissmodel.expasy.org/ (accessed on 21 October 2019), using the ArdA-H2 structure as a template found by HHblits (62.58% amino acid sequence identity and 49.00% similarity. The 3D ArdA-H1 and ArdA-H2 structures were further prepared using the Protein Preparation Wizard tool of the Maestro software [28]. In this step, all hydrogen atoms were added using PROPKA [29]. The ArdA-H1 model was further validated according to geometric and energetic properties in MolProbity [30] and QMEAN (QMEANDisCo scoring function) [27], which are available as structure assessment tools in the SWISS-MODEL web server.

**The role of the *ardA-*H1 from strain BMB9393 in gene transfer.** The *ardA*-H1 sequence was amplified using DNA (kit Wizard Genomic DNA Purification; Promega, Madison, WI, EUA) from the HA-MRSA strain BMB9393 and the primers *ard*AcompFwd/Rev (Supplementary Table S2) to provide a 574 bp amplicon. The Platinum Taq DNA Polymerase High Fidelity was used, and the PCR protocol was performed as suggested by the manufacturer (Invitrogen; Waltham, MA, USA), at a melting temperature of 55°C. The PCR product was cloned into the *E. coli* p-GEM vector-(Promega), and then transferred to the *S. aureus* vector pCN40 [31]. The pCN40::*ardA*-H1 (pACN40) and the also empty pCN40 were transferred by electroporation to *E. coli* DC10B [32], and to the CA-MRSA clinical strains 07-040 (related to USA1100 clone) and USA300-114 (related to USA300 clone) to generate 110P40A (derived from 07-040) and 300P40A (derived from USA300-114). In addition, isogenic strains (110P40E and 300P40E) that naturally lack *ardA*-H were obtained by transforming the empty pCN40 plasmid into wild type *S. aureus* strains (07-040 and USA300-114, respectively). All procedures used for the transformation were previously described by Monk and colleagues [32]. Table 1 and Table 2 describe the strains and the plasmids used and/or constructed in this study, respectively.

Clones were confirmed by PCR and Sanger sequencing, which was performed at PSEQDNA (Rio de Janeiro, RJ, Brazil). Expression of *ardA*-H1 was confirmed in the transformants by real-time qRT-PCR (Step One Real-Time PCR System (Applied Biosystem; Foster City, CA, USA)) using the Power SYBR Green RNA-to-Ct^TM^ 1-Step Kit (Applied Biosystem), as described by the manufacturer’s manual. RNA samples from the isogenic strains 07-040 and USA300-114 (*ardA*-H negatives) were also included as negative controls. For these experiments, we tested three biological replicates with three technical replicates for each. The primers used are listed in Supplementary Table S2. Table 1 and Table 2 list the strains and plasmids used in this work, respectively. The transformation experiments were performed using 1000 ng of the pLI50 shuttle vector (Addgene; Teddington, UK), recovered from *E. coli* DC10B, or 30 ng of pBMB9393 (Acc: CP005289.1), a native *S. aureus* plasmid of MRSA strain BMB9393 [1].

Electrocompetent cells were prepared [32] for the clones that expressed *ardA-*H1 (*ardA*-H1 (+)) and for the corresponding isogenic strains 110P40E and 300P40E, which naturally lack *ardA-*H (*ardA*-H (−)). The electroporation was performed exactly as described by Monk and colleagues [32]. Transformants were selected using trypticase soy broth containing 10 µg/mL of chloramphenicol (Cm10), the selectable marker of the plasmids used. Three independent biological replicates were performed with three technical replicates (colony count) for each condition. Transformation efficiency (TfE) was determined using the following formula: TfE = CFU/µDNA. Unpaired, two-tailed, Student’s *t*-test was used for statistical analysis for each pair of strains *ardA* (+) and *ardA* (−). Normality tests and *t*-tests were performed using GraphPad Prisma 9.4.

To assess whether *ardA*-H1 is expressed as dsDNA, we performed real-time qRT-PCR as described above, using cDNA from the strain BMB9393 (Acc: CP005288) that carries the *ardA*-H1 gene in ICE Tn*5801* and from the strain CR15-051, an ST30(CC30)-SCC*mec*IV MRSA, whose genome was previously sequenced by us (Acc: JAEAMC000000000) and does not carry the *ardA*-H gene.

## 4. Conclusions

Our data revealed that the two most successful and highly multidrug-resistant MRSA lineages that are widespread in humans, ST239(CC8), and livestock animals, ST398(CC398), carry *ardA*-H1 in Tn*5801* and *ardA*-H2 in Tn*916* transposons, respectively. Furthermore, our findings revealed a very strong association between the presence of these alleles and high-level multidrug resistance in randomly selected populations of *ardA*-H (−) *S. aureus*. These data were validated by the increasing in vivo DNA transfer in *S. aureus* clones of two different genetic backgrounds that expressed *ardA*-H1 and by the expression of *ardA*-H1 in the dsDNA format. Although we have not studied other elements of Tn*916* and Tn*5891*, our results demonstrated that *S. aureus* anti-restriction genes might be influential in promoting the acquisition of antimicrobial resistance genes, in addition to the tetracycline (*tetM*) gene, which is already normally carried by transposons of the Tn*916* family. Finally, investigations into the mechanisms by which bacteria overcome barriers to horizontal gene transfer, and studies of their impact on the acquisition of multidrug-resistant traits could provide important information for the design of new antibacterial strategies that may also control the accelerated evolution of multidrug-resistant bacteria.

## Figures and Tables

**Table 1:** Bacterial strains used in this study.

Strain	Description	References
*Staphylococcus aureus*		
BMB9393	ST239-SCC*mec*III, a*rdA*-H1 (+)	[1]
07-040	ST30-SCC*mec*IV, *ardA-H* (−)	[33]
110P40E	07-040 transformed with pCN40	This study
110P40A	07P40A; pACN40:*P_blaz_-ardA-*H1	This study
USA300-0114	ST8-SCC*mec*IV; *ardA*-H (−)	[34]
300P40	USA300 transformed with empty pCN40	This study
300P40A	USA300*A; pACN40:P_blaz_-ardA*-H1	This study
*Escherichia coli*		
DC10B	DC10B*∆dcm*	[32]

**Table 2:** Plasmids used in this study.

Plasmids	Description	Resistance	Reference
p-GEM T *easy* vector	Cloning vector	Amp^R^	Promega
pAGEM	*ardA-*H::pGEM	Amp^R^	This study
pCN40	Shuttle vector	Amp^R^ (*E. coli*) Ery^R^ (*S. aureus*)	[31]
pACN40	pCN40:P_blaz_-*ardA-*H1	Ery^R^	This study
pBMB9393	Native plasmid of BMB9393	Cm^R^	[1]
pLI50	Cloning vector	Cm^R^	Addgene

## Data Availability

All data are presented in this article or in the Supplementary Data. The local database containing genome sequences related to the main MLSTclonal complexes of MRSA currently circulating in the world can be accessed at http://data.mendeley.com/datasets/mkwvsp8rhg (Published on 19 April 2022).

## Supplementary Materials

The following supporting information can be downloaded at: https://www.mdpi.com/article/10.3390/antibiotics11091217/s1. Supplementary File S1. GenBank access number of the genome sequences from some selected Gram-positive bacteria carrying ardA-H1 (Tab 1) and ardA-H2 (Tab 2) used for construction of phylogenetic trees; Supplementary File S2. Assessment of resistance-associated genes acquired by horizontal transfer in the genomes carrying ardA-H1, ardA-H2, and in those lacking these genes using ResFinder 3.0; Supplementary File S3: Local database: genomes belonging to clonal complexes (CCs) commonly found among CA-MRSA, HA-MRSA and LA-MRSA; and assessment of resistance-associated genes acquired by horizontal transfer using ResFinder 3.0; Supplementary File S4. GenBank access number of the genome sequences used to construct the phylogenetic tree of the transposons Tn5801 (Tab 1) and Tn916 (Tab 2); Supplementary Table S1. Important residues described previously by Nekbyrasov et al. [19] and Mc Mahon et al. [6] for ArdA-H2 and their correspondents on ArdA-H1; Supplementary Table S2. Primers used in this study.
