# Supplementary material for: Anti-Restriction Gene Homologs Are Highly Represented in Methicillin-Resistant and Multidrug-Resistant Staphylococcus aureus ST239 and ST398: Implications for Resistance Gene Acquisitions
Source: Antibiotics (Basel). 2022 Sep 8;11(9):1217. doi: 10.3390/antibiotics11091217 (PMC9495042; doi:10.3390/antibiotics11091217)
Supplement: Supplementary file 1 [file antibiotics-11-01217-s001.zip › Supplementary Table S1.pdf]

Supplementary Table S1. Important residues described previously by Nekbyrasov *et al*. [19] and Mc Mahon *et al*. [6] for ArdA-H2 and their correspondents on ArdA-H1.

**ArdA1**	**ArdA2**	**Motif**	**Domain**
Phe92	Phe91		Central
Gln127	Ala126		
Leu128	Leu127[+]		
Gly129	Gly128		
Glu130	Glu129		
Val131	Val130		
Pro132	Pro131		
Ser133	Ala132		
Asn134	Ser133	anti-restriction	
Leu135	Leu134[+]		
Gln136	Gln135		C-terminal
Asn137	Asn136		
Tyr138	Tyr137*[+]		
Ile139	Ile138*[+]		
Asp140	Asp139[+]		
Tyr141	Tyr140		
Asp140	Asp139[+]		
Val161	Ile160	VF-motif	
Phe162	Phe161		
Tyr164	Ile163		

*Essential residues.

[+] Residues located at the dimer interface.
