# Supplementary material for: Anti-Restriction Gene Homologs Are Highly Represented in Methicillin-Resistant and Multidrug-Resistant Staphylococcus aureus ST239 and ST398: Implications for Resistance Gene Acquisitions
Source: Antibiotics (Basel). 2022 Sep 8;11(9):1217. doi: 10.3390/antibiotics11091217 (PMC9495042; doi:10.3390/antibiotics11091217)
Supplement: Supplementary file 1 [file antibiotics-11-01217-s001.zip › Supplementary Table S2.pdf]

Supplementary Table S2. Primers used in this study.

Primer	Sequence	AT*	Reference
***ard*AcompFwd**	GCATCAGAATTCAAAGGATGTGATAAGAATTGGAA	55ºC	[1]
***ard*AcompRev**	GCATCAGGATCCTAATTTATACCTCTCCGTGCTA	55ºC	[1]
**RT *ardA* fwd**	CGTCTATGTGAAGCCATTCAA	55ºC	This study
**RT *ardA* rev**	TTCCAGTAATTCTTCCAAGCTA	55ºC	This study
***cat* fwd**	GCGTGAGCTATTAAGC	55ºC	This study
***cat* rev**	GCACCCATTAGTTCAAC	55ºC	This study

*AT: annealing temperature.
